# PERSPECTIVES ON MEASURING QUALITY DEMENTIA CARE: FINDINGS FROM A STUDY IN LOW-RESOURCE LONG-TERM CARE

**DOI:** 10.1093/geroni/igae098.0572

**Published:** 2024-12-31

**Authors:** Nancy Kusmaul, Alison Rataj, Michael Lepore, Kirsten Corazzini, Yoon Chung Kim, Jing Wang, Sarah Holmes, Laura Davie

**Affiliations:** University of Maryland Baltimore County, Baltimore, Maryland, United States; University of Massachusetts Boston, Durham, New Hampshire, United States; University of Massachusetts Amherst, Amherst, Massachusetts, United States; University of New Hampshire, Durham, New Hampshire, United States; University of Maryland Baltimore, Baltimore, Maryland, United States; University of New Hampshire, Durham, New Hampshire, United States; University of Maryland School of Nursing, Baltimore, Maryland, United States; University of New Hampshire, Durham, New Hampshire, United States

## Abstract

The quality of long-term care for persons living with dementia includes elements of both quality of life and quality of care. However, which elements are most important for measuring quality is less clear and may vary based on different stakeholder perspectives. According to the National Academies of Science, Engineering, and Medicine (NASEM), quality measurement is important because it supports work towards improving quality of care, specifically making care more safe, timely, equitable, efficient, effective, and patient-centered (2022). This presentation will discuss perspectives on quality measurement among residents living with dementia, their families, staff, and administrators in low-resource long-term care settings in two states. Using a priori team coding of interviews with 59 participants, three themes emerged: (1) Quality care includes getting to know residents and communicating with them, (2) Measures of relational aspects of quality are difficult to implement, and (3) Good quality care results in better outcomes on existing clinical quality measures. During study interviews, participants readily discussed elements of quality in individual provision of care but had difficulty describing quality measurement at an organizational or systematic scale, supporting previous findings that direct observation may be an accurate indicator of quality of care, but is challenging to implement at scale. Additionally, participants conceptualized care provision from their unique perspective (i.e. staff, resident, family) suggesting that each may have different factors that constitute quality care. This study underscores the importance of quality measurement and the challenges of operationalizing quality, particularly for persons living with dementia.

